# The optimal duration of progesterone supplementation in pregnant women after IVF/ICSI: a meta-analysis

**DOI:** 10.1186/1477-7827-10-107

**Published:** 2012-12-13

**Authors:** Xi-Ru Liu, Hua-Qiao Mu, Qi Shi, Xiao-Qiu Xiao, Hong-Bo Qi

**Affiliations:** 1 Department of Obstetrics and Gynaecology, The First Affiliated Hospital of Chongqing Medical University, No.1 Youyi Road, Yuzhong District, Chongqing 400016, China; 2 Chongqing Health Centre for Women and Children, No.64 Jintang Street, Yuzhong District, Chongqing 400013, China; 3 Laboratory of Lipid & Glucose Research, The First Affiliated Hospital of Chongqing Medical University, No.1 Youyi Road, Yuzhong District, Chongqing 400016, China

**Keywords:** Progesterone, Luteal phase support, IVF/ICSI, Pregnancy outcome, Meta-analysis

## Abstract

**Background:**

Progesterone supplementation after in vitro fertilisation/intracytoplasmic sperm injection (IVF/ICSI) can improve the rates of clinical pregnancy and live birth, but the optimal duration of treatment remains controversial. The objective of this meta-analysis was to investigate the effects of early progesterone cessation on pregnancy outcomes in women undergoing IVF/ICSI.

**Methods:**

We searched MEDLINE, EMBASE, the Cochrane Central Register of Controlled Trials (CENTRAL), the Chinese biomedicine (CBM) literature database, and the Wanfang database. The final search was performed in July 2012. All available randomised trials that compared the effects of early progesterone cessation with progesterone continuation during early pregnancy after IVF/ICSI were included. The main outcome measures were live birth rate, miscarriage rate and ongoing pregnancy rate. Fixed or random-effects models were chosen to calculate the risk ratio (RR).

**Results:**

Six eligible studies with a total of 1,201 randomised participants were included in the final analysis. No statistically significant differences were detected between patients who underwent early progesterone cessation and those who received progesterone continuation for luteal phase support in terms of live birth rate (RR: 0.95, 95% CI: 0.86–1.05), miscarriage rate (RR: 1.01, 95% CI: 0.74–1.38) or ongoing pregnancy rate (RR: 0.97, 95% CI: 0.90–1.05). These results did not change after a sensitivity analysis.

**Conclusions:**

The currently available evidence suggests that progesterone supplementation beyond the first positive hCG test after IVF/ICSI might generally be unnecessary, although large-scale randomised controlled trials are needed to strengthen this conclusion.

## Background

Approximately one million couples receive in vitro fertilisation (IVF) treatment every year worldwide [1]. Luteal phase support (LPS) has routinely been applied as part of this treatment. The use of agonistic or antagonistic gonadotropin-releasing hormone (GnRH) protocols in stimulated IVF/intracytoplasmic sperm injection (ICSI) cycles cause disruptions to the luteal phase, leading to inadequate development of the endometrium and asynchrony between endometrial receptiveness and embryo transfer. The most plausible cause of this condition is the development of multiple follicles upon ovarian stimulation, which results in superphysiological steroid concentrations and consequent inhibition of luteinising hormone (LH) secretion by the pituitary via negative feedback at the level of the hypothalamic-pituitary axis [2]. Despite the rapid recovery of the pituitary in GnRH-antagonist protocols, luteolysisis also prematurely induced after GnRH-antagonist co-treatment, resulting in a significant reduction in luteal phase length and a compromised reproductive outcome. For this reason, LPS remains mandatory in GnRH antagonist protocols used for IVF [3-5]. A large number of studies have shown that LPS improves the clinical pregnancy rate and thus the live birth rate, but the ideal LPS method remains unclear [6]. Although luteal human chorionic gonadotropin (hCG) supplementation has proven to be an effective way to overcome luteal phase defects, this treatment is frequently associated with an increased risk of ovarian hyperstimulation syndrome (OHSS) [7], so the current most widely used form of LPS is progesterone (P).

The use of P supplementation after oocyte retrieval (OR) is almost universal, but the optimal duration of P administration remains controversial. A recent large survey of 84 IVF centres in 35 countries, encompassing 51,155 cycles, found that P was continued until 10–12weeks of gestation in 67% of the cycles, whereas it was discontinued in 22% and 12% when foetal heart pulsations were recognised or when the β-hCG test was positive, respectively [8]. In the existing literature, P supplementation is variously terminated on or near the day of a positive β-hCG test [9-12] or extended to the day of the first ultrasound (5–7 weeks) [13], to the 8^th^ week [14-16], or as late as the 12^th ^week of pregnancy [17-21]. Until recently, the available data have been insufficient to determine the optimal duration of therapy, and prolonged P protocols have been the rule, with most clinicians following the dictum, “better safe than sorry” [8]. A growing body of evidence, however, has challenged this concept and adds to the increasing concern that P supplementation of early pregnancy after IVF/ICSI might be unnecessary [10-14,22,23].

Four formulations of P are currently used for assisted reproduction, including vaginal, intramuscular (i.m.), oral and rectal preparations. Vaginal P was used for LPS as a single agent in 64% of cycles and in another 16% of cycles in combination with either i.m. (15%) or oral P (1%). As single agents, i.m. P was used in 13% of cycles, oral P in another 2% and hCG in 5% [8]. Vaginal P can result in similar pregnancy rates as i.m. P and is more comfortable and tolerable to patients [24,25], but it is more expensive. Conversely, i.m. P is often associated with a number of side effects, including painful injections, severe inflammatory reactions, and sterile abscesses [26]. Prolonged and repeated i.m. injections of P in oil may also lead to delayed forms of hypersensitivity reactions, with leukocytosis, marked eosinophilia and compromised pulmonary activity [27,28]. Orally administered P has a first-pass effect in which a high concentration is sent to the portal circulation, which, in turn, results in the production of many liver metabolites of P, some of which may be teratogenic. Despite the available literature on the teratogenic effects of prenatal oral P use [29,30], this agent is still used routinely by many practitioners. Therefore, taking into consideration the burden of LPS treatment, the adverse reactions to P and updated results suggesting that P supplementation during early pregnancy after IVF/ICSI might be unnecessary, we questioned whether the practice of early pregnancy P supplementation in IVF/ICSI patients should be discontinued.

The aim of this study was to perform a meta-analysis of all available randomised controlled trials (RCT) comparing early P cessation with P continuation after assisted conception in IVF/ICSI cycles to investigate potential differences in live birth, miscarriage and ongoing pregnancy rates. This review was performed in accordance with the preferred reporting items for systematic reviews and meta-analyses (PRISMA) statement principles [31].

## Methods

### Types of studies

The inclusion criteria for eligible studies were defined a priori during the design phase of this systematic review. Randomised controlled trials investigating the duration of P supplementation for luteal phase support in IVF/ICSI cycles were included. Trials using donor oocyte cycles or frozen transfers were excluded. No limitations were placed on language, date, or publication status.

### Types of participants

Women undergoing IVF/ICSI who were evaluated for the effects of P supplementation duration on pregnancy outcomes were included.

### Types of interventions

The interventions evaluated were early P cessation versus P continuation during the first trimester in pregnant women after IVF/ICSI.

### Types of outcome measures

The primary outcome chosen for the meta-analysis was live birth rate (LBR, i.e.,a baby born alive after 24 weeks gestation). Secondary outcomes included ongoing pregnancy rate (OPR, pregnancy beyond 12 weeks of gestation, as confirmed by foetal heart activity on an ultrasound), and miscarriage rate (MR, the failure to achieve live birth after a positive β-hCG test).

### Literature search and data collection

We performed an exhaustive electronic search in the following databases: MEDLINE (1946 to July 2012), EMBASE (1974 to July 2012), the Cochrane Central Register of Controlled Trials (CENTRAL), the Chinese biomedicine (CBM) literature database (1978 to July 2012), and the Wanfang database (1998 to July 2012). The search combined terms and descriptors related to IVF, ICSI, luteal phase support, and progesterone. To fit with the syntax used in each consulted database, the search strategy was modified with a series of terms suggestive of RCTs as set out by the Cochrane Handbook for Systematic Review of Intervention [32](Additional file 1). No limit was placed on language. We also carefully browsed the references of relevant publications and added the related publications to the search. When questions related to the design or outcomes of the trials arose, we contacted the corresponding authors to confirm the information we extracted from their trials or to clarify any ambiguities.

### Assessment of the risk of bias in the included studies

The risk of bias in the included studies was assessed independently by two reviewers (Xi-Ru Liu and Hua-Qiao Mu) according to the guidelines recommended in the Cochrane Handbook for Systematic Review of Intervention [32]. For each study, we assessed the risk of bias related to sequence generation, allocation, blinding of participants and personnel, blinding of outcome assessment, incomplete outcome data, selective reporting and other sources of bias. A judgment of ‘Yes’ meant a low risk of bias, a judgment of ‘No’ meant a high risk of bias, and ‘Unclear’ indicated an unclear risk of bias. Disagreements were discussed and resolved by consensus with a third reviewer (Qi Shi).

### Data extraction and synthesis

Data extraction was performed independently by two reviewers (Xi-Ru Liu and Hua-Qiao Mu). Discrepancies were resolved by discussion with a third reviewer (Qi Shi).We extracted the following information from each eligible study: first author, year of publication, country of origin, sample size, and a number of patient characteristics, including the IVF protocol used, the exact dose of P, the route of administration, the timing of initiation and duration of luteal phase support with P, and IVF/ICSI outcomes.

Raw data were extracted from the eligible studies for each defined outcome and pooled using Review Manager 5.1 software. Dichotomous results from each study were expressed as relative risk (RR) with 95% confidence intervals (CI). These results were combined for the meta-analysis using a fixed-effects model in the absence of statistically significant heterogeneity or a random-effects model in the presence of statistically significant heterogeneity. The inter-study heterogeneity was evaluated using the *x*^2 ^(Cochran's Q) statistic and the *I*^2 ^value. Sensitivity analyses were performed for those studies that answered the research question of interest but used a quasi-randomised approach for patient allocation. Subgroup analyses were planned a priori based on: (1) the timing of randomisation, (2) the timing of initiation of P, (3) the GnRH analogue used for LH surge inhibition, and (4) the type and dose of P administration.

## Results

### Literature search results

**Figure 1:**
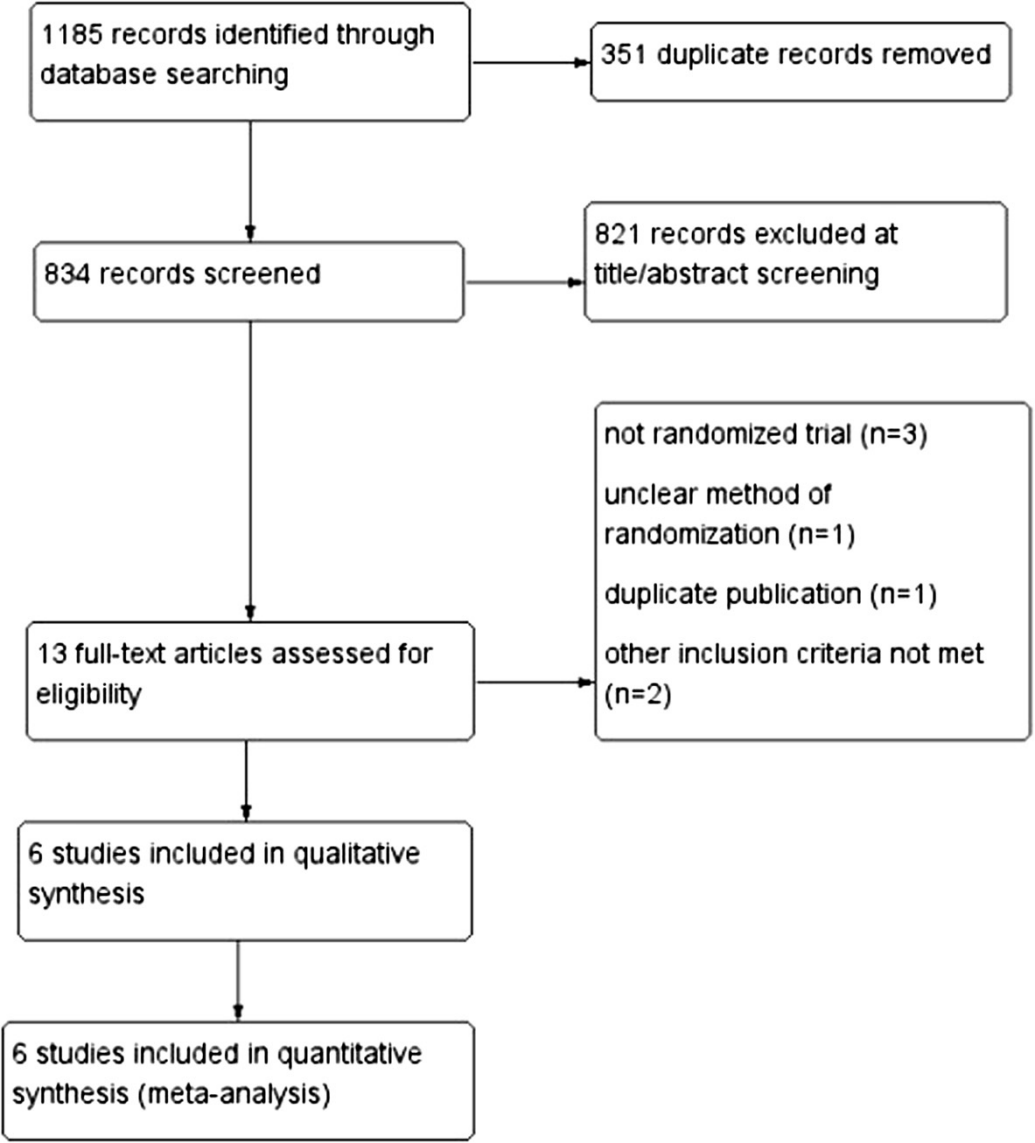
Flow diagram of the systematic review and meta-analysis.

A total of 1,185 trials were retrieved in the initial electronic search, 351 of which were duplicate records that were subsequently removed. An additional 821 were excluded upon title/abstract screening. The 13 remaining trials were selected for further full-text analysis. Seven of these trials were excluded. Two were retrospective cohort studies [10,22], and one failed to implement randomisation [33]; one was the same study reported as an abstract at an earlier meeting [34]; one trial did not explicitly describe the sequence generation or allocation concealment [35]; and two trials did not meet other inclusion criteria [18,36]. The remaining six RCTs, totalling 1,201 participants, were included in this meta-analysis. Detailed search procedures are summarised in the flow diagram (Figure 1).

### The methodological quality of included studies

**Figure 2:**
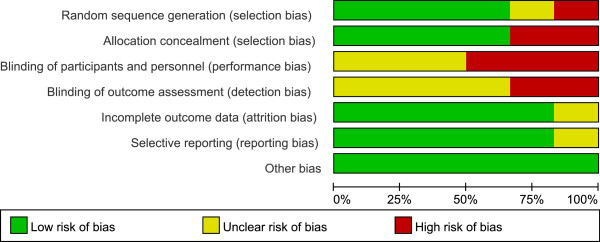
The risk of bias in the included studies.

**Figure 3:**
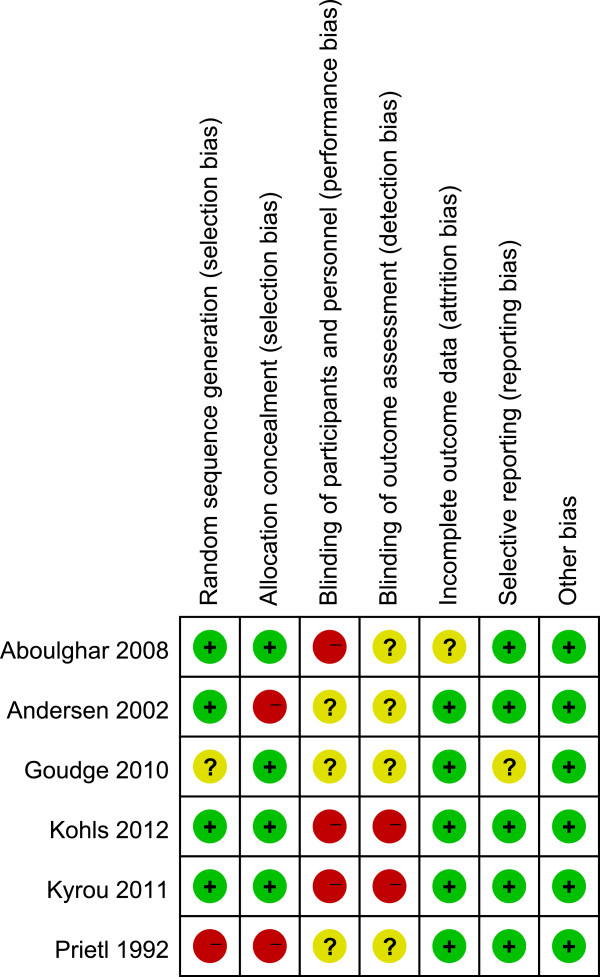
Summary of the risk of bias in the included studies.

Four of the six trials provided an adequate randomisation model [11,13,14,23], and four an adequate mode for allocation concealment [12-14,23], all of which made use of sealed and opaque envelopes. One trial [17] used odd and even patient birth years for allocation and was classified as a quasi-randomised trial. Only one of the studies was a dual centre study [11]; the other five were unicentric. None of the studies blinded their personnel, participants or outcome assessors or at least did not mention blinding. Owing to the small number of included studies, it was impossible to conduct a meaningful assessment of publication bias using a funnel plot. See the risk of bias graph (Figure 2) and risk of bias summary (Figure 3) for an overview.

### Characteristics of included studies

The six selected studies [11-14,17,23] were performed in Spain, Belgium, Minnesota (USA), Egypt, Denmark, and Germany, respectively, and involved 1,201 participants who were originally studied between 1989 and 2010. In two studies, patients with a clinical pregnancy (at 5–7 weeks of gestation) were included [13,14], and in three studies, patients with a positive β-hCG test (on the 11-16^th ^day post-embryo transfer (ET)) were included [11,17,23]. In the final study, patients were enrolled at the beginning of an IVF cycle [12]. The type and dose of P supplementation, the timing of administration and the duration of P supplementation varied among the studies. In addition, controlled ovarian hyperstimulation (COH) protocols and the basal clinical characteristics of the patients differed between studies. These data are presented in Table 1.

**Table 1:** Characteristics of the studies included in this review

**Study Author, year**	**Timing of randomisation**	**ART**	**COH protocols**	**Total**	**Initiation of P**	**Dose & route of administration**	**No.**	**Early P cessation group**	**No.**	**Continuation group**
Kohls, 2012	Clinical pregnancy	IVF/ICSI	GnRH-anta	220	OR	vaginal P 200mg bid	110	week 5	110	week 8
Kyrou, 2011	Positive hCG test	IVF/ICSI	GnRH-anta	200	ET	vaginal P 200mg tid	100	the 16^th ^day post-ET	100	week 7
Goudge, 2010	COH	IVF	GnRH-a/GnRH-anta	101	ET/OR	IM P50mg qd	53	the 11^th ^day post-ET	48	week 6
Aboulghar, 2008	Clinical pregnancy	ICSI	GnRH-a	257	Unstated	IM or vaginal P	125	week 6-7	132	week 9-10
Andersen, 2002	Positive hCG test	IVF/ICSI	GnRH-a	303	ET	vaginal P 200mg tid	150	the 14^th ^day post-ET	153	week 7
Prietl, 1992	Positive hCG test	IVF	CC/hMG/GnRH-a	120	Unstated	PC500mg/EV10mg tiw	65	the 12^th ^day post-ET	55	week 12

### Live birth rate

**Figure 4:**
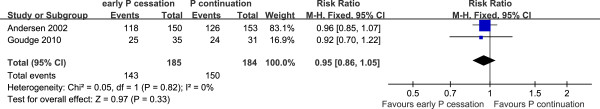
Live birth rate of women who underwent early P cessation versus P continuation after IVF/ICSI.

Two eligible studies presented data on live birth rates [11,12]. In the study reported by Goudge et al. [12], the number of patients recruited at the beginning of an IVF cycle was converted to the number of patients with a positive β-hCG test according to the reported biochemical pregnancy rate. There were 143 events in the early P cessation group (in which P was stopped on the 11^th ^or 14^th ^day post-ET) and 150 in the P continuation group (in which P was continued until the 6^th ^or 7^th^ week of gestation). There were a total of 293 patients who gave birth to live babies out of 369 participants. The probability of live birth did not differ between the early P cessation group (77.3%, 143/185) and the P continuation group (81.5%, 150/184) (P = 0.33; RR: 0.95, 95% CI: 0.86–1.05). There was no statistical heterogeneity in this comparison (χ^2 ^= 0.05, df = 1, P = 0.82; I^2^ = 0%) (Figure 4).

### Miscarriage rate

**Figure 5:**
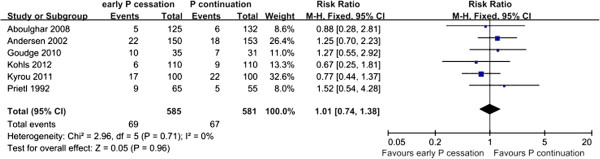
Miscarriage rate of women who underwent early P cessation versus P continuation after IVF/ICSI.

MR data were available from six studies, with 136 events out of 1166 participants; after data conversion, this figure corresponded to 69/585 in the early P cessation group and 67/581 in the P continuation group [11-14,17,23]. No statistical heterogeneity was observed between the studies (χ^2^ = 2.96, df = 5, P = 0.71; I^2^ = 0%). There were no significant differences in the number of miscarriages between patients who received early P cessation and those who received P continuation (P = 0.96; RR: 1.01, 95% CI: 0.74–1.38) (Figure 5).

### Ongoing pregnancy rate

**Figure 6:**
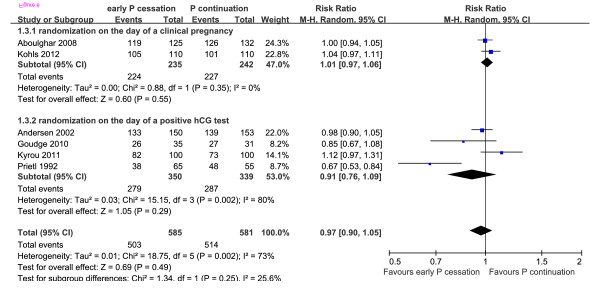
Ongoing pregnancy rate of women who underwent early P cessation versus P continuation after IVF/ICSI.

OPR data were available from six studies, with 1017 events among 1166 participants (503/585 in the early P cessation group and 514/581 in the P continuation group) [11-14,17,23]. A meta-analysis of all six trials yielded an RR of 0.97 (P = 0.49; 95% CI: 0.90–1.05), indicating that there was no statistically significant difference between the early P cessation and P continuation groups (Figure 6).

Because the OPR was heterogeneous (χ^2 ^= 18.75, df = 5, P = 0.002; I^2 ^= 73%) among the included studies, a random-effects model was used. No differences were observed between the results obtained using a fixed-effects model (P = 0.28; RR: 0.98; 95% CI: 0.94–1.02) and those obtained from the random-effects model (P = 0.49; RR: 0.97; 95% CI: 0.90–1.05), although the weight of each study was altered. Next, according to the timing of randomisation, we performed a subgroup analysis and separately pooled four studies [11,12,17,23] in which P was withdrawn on the day of a positive β-hCG test and two studies [13,14] in which P was withdrawn on the day that clinical pregnancy was confirmed (5^th^–7^th^ weeks of gestation). This stratified analysis revealed no significant differences between the groups in which P was stopped on the day of a positive β-hCG test (P = 0.29; RR: 0.91; 95% CI: 0.76–1.09) or on the day that clinical pregnancy was verified (P = 0.55; RR: 1.01; 95% CI: 0.97–1.06). Heterogeneity was detected in the subgroup of studies that randomised patients on the day of a positive β-hCG test (χ^2 ^= 15.15, df = 3, P = 0.002; I^2 ^= 80%). The study reported by Prietl [17] might be the source of heterogeneity, as it used a different luteal phase support protocol (17α-hydroxyprogesterone caproate (100mg) and oestradiol valerate (10mg) twice a week) and exhibited a high risk of bias based on sequence generation and patient allocation methods. In a sensitivity analysis, we recalculated the combined results while excluding this study. However, the results before (P = 0.49; RR: 0.97; 95% CI: 0.90–1.05) and after the sensitivity analysis (P = 0.74; RR: 1.01; 95% CI: 0.96–1.06) were not significantly different.

## Discussion

Although there is no firm evidence to support the continuation of LPS until the 10^th^ to 12^th^ week of gestation, this practice is used in the majority of IVF cycles worldwide [8]. This review compared the effects of early cessation with continuation of P supplementation for luteal phase support in pregnant women after IVF/ICSI, focusing on the live birth, ongoing pregnancy and miscarriage rates. The pooled results showed no significant differences in LBR between groups in which P supplementation was stopped on the day of a positive β-hCG test or for whom P supplementation was continued up to the 6^th^ to 7^th ^week of gestation. Similarly, the miscarriage and ongoing pregnancy rates were not affected by the duration of P administration. Because there was statistical heterogeneity in the studies analysed for OPR, we performed a subgroup analysis and a sensitivity analysis. The results of the subgroup analysis were in accordance with the above results. The findings were also stable after the sensitivity analysis, which excluded one study [17] in which odd or even patient birth years were used for patient allocation. Based on this analysis, we find no convincing evidence to support the routine use of P supplementation during early pregnancy in women undergoing IVF/ICSI. It is possible that the establishment of a pregnancy and rescue of the corpus luteum via trophoblastic hCG may make up for the possible luteal phase defect caused by the stimulated IVF cycles.

Most of the studies included in this review described their methods of sequence generation and allocation concealing. However, none of the studies mentioned blinding. Keeping trial participants, personnel, or assessors blinded to the assigned intervention might reduce the influence of subjective psychological factors on pregnancy outcomes, an important aspect of RCTs. However, owing to the nature of current LPS studies, absolute double blinding is often not practical, as it is not possible to blind the participants. None of the studies explicitly mentioned blinding of personnel or outcome assessors. Nevertheless, it is unlikely that pregnancy outcomes such as live birth, miscarriage or ongoing pregnancy can be affected by detection bias. In future studies, proper blinding protocols using a double-dummy design should be implemented, and a placebo control group should be established.

Due to the small number of studies that met the inclusion criteria and the different clinical characteristics of the participants, it was impossible to conduct meaningful subgroup analyses based on the initiation of P supplementation, the GnRH analogue used for luteinising hormone surge inhibition, or the type or dose of P administration. These analyses might become practical upon the accumulation of further studies. We were only able to analyse studies according to the different timing of randomisation, a potential source of clinical heterogeneity; here, we pooled the data from studies with similar enrolment designs. However, it should be noted that the aforementioned parameters do vary among the included studies, and we do not know whether these clinical variables might be associated with the effects of P supplementation on pregnancy outcomes. For example, P supplementation was initiated at different time-points in these studies. These were no significant differences between initiation on the day of OR or on the day of ET with regard to clinical pregnancy, ongoing pregnancy, or live birth rates, according to recent reports [36,37]. One study suggested that delaying the LPS until six days after OR can decrease the pregnancy rate [38]. Because P administration was initiated on the day of OR or ET in most of the eligible studies in our analysis, it is unlikely that these discrepancies affected our results. In a word, our results require confirmation in further studies in which the baseline of different groups is comparable to the greatest possible extent.

Two important limitations of our meta-analysis should be noted: (1) only six studies were included in this review, and the number of patients analysed is far below the sample size required to exclude a clinically important difference. A non-inferiority trial showing a difference of −4% or larger from a live birth rate of 80% would require a sample size of 3,140 women with a positive pregnancy test after IVF [39]. (2) The external validity of the study may be limited because existing studies excluded those patients with early bleeding, advanced age or polycystic ovary syndrome as well as those patients with an inadequate hCG rise or endometriosis, whose luteal phase may behave differently. Future trials should recruit such patients to better stratify the outcomes for these patient groups.

## Conclusions

In conclusion, based on the currently available evidence, progesterone supplementation might be unnecessary beyond the first positive β-hCG test after IVF/ICSI. However, considering the large number of IVF cycles performed globally and the side effects and costs of progesterone treatment, additional well-designed RCTs are urgently needed to investigate the optimal duration of progesterone administration during early pregnancy in women undergoing IVF/ICSI.

## Abbreviations

COH: Controlled ovarian hyperstimulation; ET: Embryo transfer; GnRH: Gonadotropin-releasing hormone; hCG: human chorionic gonadotropin; IVF: In vitro fertilisation; ICSI: Intracytoplasmic sperm injection; i.m: intramuscular; LBR: Live birth rate; LH: Luteinising hormone; LPS: Luteal phase support; MR: Miscarriage rate; OR: Oocyte retrieval; OPR: Ongoing pregnancy rate; P: Progesterone; RCT: Randomised controlled trials.

## Competing interests

The authors declare that they have no competing interests.

## Authors’ contributions

Hong-Bo Qi was responsible for designing and coordinating the study. All authors were responsible for data collection, data analysis, and data interpretation. Xi-Ru Liu, Hua-Qiao Mu, and Qi Shi were responsible for the statistical analysis and for writing the manuscript. Xiao-Qiu Xiao was responsible for reviewing the manuscript. All authors read and approved the final manuscript.

## Supplementary Material

Additional file 1 Search strategy in MEDLINE, EMBASE, and CENTRAL databases. Click here for file
