# Supplementary material for: The optimal duration of progesterone supplementation in pregnant women after IVF/ICSI: a meta-analysis
Source: Reprod Biol Endocrinol. 2012 Dec 13;10:107. doi: 10.1186/1477-7827-10-107 (PMC3551800; doi:10.1186/1477-7827-10-107)
Supplement: Additional file 1 — Search strategy in MEDLINE, EMBASE, and CENTRAL databases. [file 1477-7827-10-107-S1.pdf]

Additional file 1 Search strategy in MEDLINE, EMBASE, and CENTRAL databases

MEDLINE search strategy

1 exp embryo transfer/ or exp fertilization in vitro/ or exp sperm injections, intracytoplasmic/ (30096)

2 embryo transfer$.tw. (7407)

3 in vitro fertili?ation.tw. (15310)

4 ivf-et.tw. (1717)

5 (ivf or et).tw. (163058)

6 icsi.tw. (4801)

7 intracytoplasmic sperm injection$.tw. (4423)

8 (blastocyst adj2 transfer$).tw. (462)

9 or/1-8 (185938)

10 exp Luteal Phase/ (4337)

11 (luteal adj5 support$).tw. (486)

12 (luteal adj5 phase).tw. (8144)

13 (ischemic adj5 phase).tw. (824)

14 post ovulat$.tw. (621)

15 (post adj5 transfer$).tw. (918)

16 (after adj5 transfer$).tw. (15621)

17 (post adj5 trigger$).tw. (303)

18 (after adj5 trigger$).tw. (2163)

19 or/10-18 (29831)

20 9 and 19 (3730)

21 exp Progesterone/ (61387)

22 Progesterone$.tw. (63769)

23 dydrogesterone.tw. (333)

24 utrogest.tw. (4)

25 17 alpha-hydroxyprogesterone.tw. (1170)

26 Prontogest.tw. (5)

27 crinone.tw. (46)

28 or/21-27 (90227)

29 20 and 28 (811)

30 randomized controlled trial.pt. (332880)

31 controlled clinical trial.pt. (84709)

32 randomized.ab. (248208)

33 placebo.ab. (138036)

34 drug therapy.fs. (1552677)

35 randomly.ab. (181912)

36 trial.ab. (257439)

37 groups.ab. (1185300)

38 or/30-37 (2979602)

39 exp animals/ not humans.sh. (3758218)

40 38 not 39 (2544493)

41 29 and 40 (322)

EMBASE search strategy

#1 'embryo transfer'/exp OR 'embryo transfer' OR 'fertilization in vitro'/exp OR 'fertilization in vitro' OR 'intracytoplasmic sperm injection'/exp OR 'intracytoplasmic sperm injection' (47,731)

#2 'in vitro fertili?ation' OR 'ivf et' OR ivf OR et OR 'icsi'/exp OR icsi (1,323,016)

#3 'embryos transfer' OR 'embryo transfers' OR 'intracytoplasmic sperm injections' (1,212)

#4 blastocyst NEAR/2 transfer (723)

#5 #1 OR #2 OR #3 OR #4 (1,337,754)

#6 'luteal phase'/exp OR 'luteal phase' (11,807)

#7 luteal NEAR/5 support* (687)

#8 luteal NEAR/5 phase (11,946)

#9 ischemic NEAR/5 phase (1,106)

#10 post AND ovulat* (2,979)

#11 post NEAR/5 transfer* (1,298)

#12 after NEAR/5 transfer* (19,276)

#13 post NEAR/5 trigger* (424)

#14 after NEAR/5 trigger* (2,644)

#15 #6 OR #7 OR #8 OR #9 OR #10 OR #11 OR #12 OR #13 OR #14 (38,995)

#16 #5 AND #15 (6,804)

#17 'progesterone'/exp (80,832)

#18 progesterone* OR 'dydrogesterone'/exp OR dydrogesterone OR trogest OR 17 AND 'alpha hydroxyprogesterone' OR 'prontogest'/exp OR prontogest OR 'crinone'/exp OR crinone (81,099)

#19 #17 OR #18 (81,099)

#20 'randomized controlled trial'/exp OR 'randomized controlled trial' (343,986)

#21 'clinical trial controlled'/exp OR 'clinical trial controlled' (423,798)

#22 randomized:ab (314,193)

#23 placebo:ab (175,604)

#24 randomly:ab (223,200)

#25 trial:ab (334,761)

#26 groups:ab (1,551,859)

#27 #20 OR #21 OR #22 OR #23 OR #24 OR #25 OR #26 (2,246,373)

#28 #16 AND #19 AND #27 (578)

CENTRAL search strategy

1 exp embryo transfer/ or exp fertilization in vitro/ or exp sperm injections, intracytoplasmic/ (1573)

2 embryo transfer$.tw. (878)

3 in vitro fertili?ation.tw. (1312)

4 ivf-et.tw. (253)

5 (ivf or et).tw. (6064)

6 icsi.tw. (647)

7 intracytoplasmic sperm injection$.tw. (405)

8 (blastocyst adj2 transfer$).tw. (64)

9 or/1-8 (7236)

10 exp Luteal Phase/ (423)

11 (luteal adj5 support$).tw. (211)

12 (luteal adj5 phase).tw. (859)

13 (ischemic adj5 phase).tw. (112)

14 post ovulat$.tw. (12)

15 (post adj5 transfer$).tw. (45)

16 (after adj5 transfer$).tw. (5923)

17 (post adj5 trigger$).tw. (11)

18 (after adj5 trigger$).tw. (2089)

19 or/10-18 (8895)

20 9 and 19 (1465)

21 exp Progesterone/ (2129)

22 Progesterone$.tw. (2202)

23 dydrogesterone.tw. (146)

24 utrogest.tw. (7)

25 17 alpha-hydroxyprogesterone.tw. (71)

26 Prontogest.tw. (5)

27 crinone.tw. (37)

28 or/21-27 (3548)

29 20 and 28 (268)
